# Influence of training and selection on postural stability and its relationship with sport level in judo practitioners aged 11–14 years

**DOI:** 10.3389/fpsyg.2022.1053426

**Published:** 2023-01-06

**Authors:** Janusz Jaworski, Grzegorz Lech, Kazimierz Witkowski, Przemysław Bujas, Katarzyna Szczepanik, Paweł Piepiora

**Affiliations:** ^1^Faculty of Physical Education and Sport, University of Physical Education in Krakow, Kraków, Poland; ^2^Faculty of Physical Education and Sports, Wrocław University of Health and Sport Sciences, Wrocław, Poland

**Keywords:** combat sports, balance, somatosensory science, children and adolescents, postural stability (postural control)

## Abstract

The aim of this study is to determine the influence of training and selection on postural stability and its relationship with the sports level of judo practitioners aged 11–14 years. The study group consisted of 21 children judokas, aged 11–12, and 80 of their non-training peers, as well as 19 adolescent judokas, aged 13–14, and 76 of their non-training peers. The judokas were surveyed during regional championships. The level of achievement was determined by the place taken in the tournament. The balance was assessed with the use of a CQ Stab 2P stabilographic platform (CQ Elektronik System, Poland). The device recorded the position of the foot center of pressure (COP) from 6 sensors; 3 of them being located in each platform plate. The following parameters describing the movement of the foot COP were analyzed: total path length calculated in both axes (SP); mean COP inclination (MA), size of the surface area delineated by COP (SA); mean COP frequency (MF). Significantly higher values of SP, MA, and SA were noted in non-training children (aged 11–12), while MF values were significantly higher in young judokas. The same regularity was found in the older age group. Upon comparing the means between children judokas and adolescent judokas, significant differences were noted in the case of SP and MF. In both cases, higher mean values were found in the younger judoka group. A similar comparison in the non-training group indicates statistically significant better values of all analyzed indicators in the 13–14 year-old group, except for MF. Upon examining the relationship between the values of the parameters characterizing the balance level and the sports level, both in the group of training children and adolescents, insignificant values of correlation coefficients were obtained.

## Introduction

Balance, understood as the process of controlling the location of the body’s gravity projections in the area of the base area of the body, takes its most complex form in situations requiring resistance to strong destabilizing stimuli (Kioumourtzoglou et al., 1997; Blaszczyk et al., 2000; Paillard and Noe, 2006). Conceptually, it approaches the phenomenon of stability, describing the physiological mechanisms of maintaining and restoring posture that prevent falling. A fall is caused by a person’s center of mass (COM) going beyond the stability limit, defined as the optimal position of the foot center of pressure (COP) within the base surface and the size of the safety limit (Blaszczyk et al., 2000; Blaszczyk, 2010). Postural stability is important in everyday situations, but it is in sport that its maintenance requires the most efficient regulatory and control processes (Hrysomallis et al., 2006; Paillard and Noe, 2006). Most studies show a strong positive relationship between physical activity, including sports, and the improvement of balance indicators. Comparisons between training people practicing judo, karate and wrestling, and non-training people, seem to confirm this thesis (Hrysomallis, 2011; Rzepko et al., 2019; Slomka et al., 2019). Nevertheless, the research also shows that such an advantage is not achieved in all conditions of the implementation of balance maintaining task. In the absence of visual control, the authors report a lack of differences between the training and the healthy non-training people (Paillard and Noe, 2006) in terms of balance indices.

Assuming that a smaller range of swaying is synonymous with a higher level of balance in non-training individuals, it should be remembered that this indicator, very often determined by researchers basing on measurement in static conditions, does not reflect the complexity of individual strategies for controlling body stability developed by athletes from various sport disciplines (Blaise Williams et al., 2016). All the more so if the sports activity requires its dynamic form. The authors’ opinions indicating that increased swaying while standing calmly is not always synonymous with postural instability, as the control of spontaneous swaying and postural response to balance disturbances are carried out on the basis of various posture control mechanisms (Blaszczyk, 2010). These mechanisms are particularly important in sport disciplines where the goal is to disrupt the opponent’s stability and make him/her fall. Judo is such a sport. The essence of stand-up fighting in this discipline is to throw the opponent off balance by leaning (Japanese *kuzushi*) and then by stepping forward and throwing (Japanese *kake*) causing him/her to fall onto the largest possible area of the back. Judo fighting is based on the constant throwing not only the opponent, but also oneself off balance, as it is an indispensable condition for an effective throw. Loss of balance, just like regaining it, are permanent states during a fight, as 70% of the fight takes place in an upright position (Sterkowicz and Franchini, 2000).

Maintaining balance is based on the broad integration of the activities of various human body systems: vestibular, visual, somatosensory, and motor systems (Nashner, 1997). The functions of the sensory systems ensure the control of head movements in space, the movement of individual body segments in relation to each other or contact with external objects (Winter, 1995; Ivanenko and Gurfinkel, 2018). Their hierarchy changes depending on the conditions in which the stability control processes must take place. The reference to this information is due to the fact that what remains unresolved in the case of judokas, is their stability. In different sport disciplines, depending on their specificities, the role of visual, vestibular, or somatosensory information may increase. The authors (Blaise Williams et al., 2016) also suggest that whether the athletes train or compete on a stable or unstable ground will result in the development of specific motor strategies in response to destabilizing factors. In combat sports, and judo in particular, the role of vestibular information is assumed to be important: both from within the lower limbs joints, which seems obvious to maintain balance, but also from the upper limbs and the torso.

Studies conducted using computerized dynamic posturography (CDP) by Leong et al. (2011) show significantly better balance of taekwondo athletes, mainly in static tests with eyes closed. This indicates a significant share of the somatosensory base in combat sports athletes. This type of research makes it possible to assess changes in the structure of balance control, and the fact that the aforementioned work analyzed the results of beginner yet adult athletes suggests that training during the developmental period may bring even greater benefits, overlapping with natural development processes.

The analysis of these natural changes underlying postural stability shows that they occur in different ways depending on the type of motor task. This involves a response based on proprioceptive, vestibular, or visual mechanisms, which changes with individual development. The age of our subjects, compared to younger children, is conducive to the improvement of stability indicators based primarily on somatosensory information and suppression of incorrect visual information. In older age categories, progressive changes concern mainly vestibular-based responses (Steindl et al., 2006).

So far, the inquiries of scientists have focused mainly on the analysis of changes in the level of stability along with the increasing training experience. A less frequently investigated field, however, is the relationship between the level of balance presented by athletes and their sports level, i.e., the research on determining the role of balance and its impact on the sports result. In studies carried out in the groups of cadets (aged 15–16), juniors (aged 16–18), and seniors (aged over 18) (Lech et al., 2007, 2011, 2014), no correlation was found between the level of balance (assessed using the Flamingo balance test) and the sports level of athletes. When examining contestants aged 16–19, divided into two categories in terms of their sports level, Paillard et al. (2002) did not observe any significant differences between the stability indices under static conditions, indicating only a different sensory organization between the groups. This implies that specific forms of motor control were developed during training that distinguished better athletes from worse athletes.

The novelty of the present is the analysis of the impact of training and selection on balance indices in judo practitioners aged 11–14. No results of analyzes on this subject were found in the available literature. In addition, the relationship between these indicators and the level of achievement in occupations was examined. Also this topic is completely new to this age group.

The authors of the study adopt a working hypothesis that targeted judo training increases the level of stability control, and its level depends on the level of sports achievements.

## Materials and methods

### Participants

The study group consisted of 21 children judokas aged 11–12 and 80 of their non-training peers, and 19 adolescent judokas aged 13–14 and 76 of their non-training peers. Only healthy individuals without any short-term or long-term injuries were qualified for the study. The judoka tests were carried out during regional championships. They were made on the competition day, after the official weigh-in. The comparative group consisted of students from schools in the Małopolskie province in Poland. They did not participate in any organized physical activity, either now or in the past. The tests in all groups were carried out in a separate room ensuring peace and quiet, the ambient temperature was 22°C; they took place in the morning (10–12) and were conducted by the same research team.

Participation in the research was voluntary. It was carried out in accordance with the Helsinki Declaration. Each subject was informed about the purpose of the research and could withdraw from further participation in the research at any time without giving any reason. The coach, who was the legal guardian of the subject during the competition, and, in the case of the comparison group, the parents and the school principal had to give their consent to the study participation.

Body weight was determined using the Tanita TBF-551 body composition analyzer (Japan) with an accuracy of 0.1 kg. Body height (BH) was measured with an accuracy of 0.1 cm according to Martin’s technique, using a set of Swiss devices from SIEBER HEGNER MACHINES SA. The research also used a questionnaire, which included questions about age and training experience. The level of achievement was determined based on the place taken in the tournament. Thus: 1st place–9; 2nd–7; 3rd–5; 5th–3.5; 7th–1.5; 9th–0.5 points. Tables 1, 2 show the characteristics of age, training experience, and somatic indicators of the study participants in two age categories.

**TABLE 1:** Age, training experience, and somatic build of study participants in children’s group (age 11–12).

	Children judokas					Non-training peers				
	*N*	Mean	Min.	Max.	*SD*	*N*	Mean	Min.	Max.	*SD*
Age (years)	21	11.4	11.0	12.0	0.5	80	11.6	11.0	12.0	0.5
Height (cm)	21	149.3	141.0	164.0	5.8	80	154.7	141.0	177.0	8.0
Body mass (kg)	21	40.6	30.3	47.8	5.4	80	48.0	28.9	90.1	12.2
Training experience (years)	21	4.3	3.0	7.0	1.7	0				
Level of achievement (points)	21	3.9	0.0	9.0	3.0	0				

**TABLE 2:** Age, experience, and somatic build of study participants in the group of adolescents (13–14 years old).

	Adolescent judokas					Non-training peers				
	*N*	Mean	Min.	Max.	*SD*	*N*	Mean	Min.	Max.	*SD*
Age (years)	18	13.4	13.0	14.0	0.5	76	13.3	13.0	14.0	0.5
Height (cm)	18	163.6	149.0	176.5	6.9	76	162.7	139.0	182.0	8.83
Body mass (kg)	18	54.8	43.1	74.7	8.3	76	53.8	30.8	95.1	13.1
Training experience (years)	18	5.8	4.0	8.0	1.3	0				
Level of achievement (points)	18	2.8	0.0	9.0	3.1	0				

### Study design

The balance was assessed on the CQ Stab 2P stabilographic platform (CQ Elektronik System, Poland). The device recorded the position of the foot COP from 6 sensors, located 3 in each platform plate. Sampling was 200 Hz for each sensor. The platform plates were properly leveled and their surfaces their surfaces were aligned to form a single plane. The study consisted of a 30 s measurement of body stability in a relaxed standing position with eyes open. The width of the lower limb spacing and the angle of the feet opening were natural, unconstrained. Opposite the test subject, a fixation point was placed 1 m away. Once on the platform, the subject stood still and tried to keep his/her gaze on the fixation point. Participants did not perform a warm-up or practice test. The following parameters describing the movement of the foot pressure center (COP) were analyzed:

1. SP (mm) (Sway Path)—the total length of the path in both axes;
2. MA (mm) (mean amplitude)—mean sway of COP (foot pressure center);
3. SA (mm^2) (Sway Area)—size of the surface area delineated by the COP;
4. MF (Hz) (Mean Frequency)—mean frequency of the COP.

The study was approved by Regional Medical Chamber in Kraków, Poland, and granted permission No. 108/KBL/OIL/2014.

### Statistical analysis

Multivariate tables were used to characterize the study material. For comparisons of means, the Student’s *t*-test for unrelated variables, the Cochran–Cox test and the Mann–Whitney U test were used, depending on the distribution and homogeneity of variance. The homogeneity of variance was checked with Levene’s test. The assumption of normality of distributions was verified by the Shapiro–Wilk W test. Spearman’s rank correlation coefficient (R) according to the variable type was used in the correlation analysis. A significance level of *p* < 0.05 was adopted in all analyzes. STATISTICA 13.3 software was used for statistical analysis.

## Results

Table 3 summarizes the values of the parameters characterizing the level of balance in judo children (11–12 years old) and their non-training peers. When comparing the mean values between the studied groups, statistically significant differences were obtained in all cases. For SP (mm), MA (mm), and SA (mm^2), higher mean values were recorded in the non-training, respectively: 343.09, 2.71, and 303.16. In judo athletes, these averages were respectively, 313.10, 1.23, and 113.43. In the MF (Hz) range, judokas had a higher mean frequency of corrective reactions (1.69, in non-trainers: 0.77).

**TABLE 3:** Values of parameters characterizing the level of balance in children judokas (11–12 years old) and their non-training peers.

	Children judokas					Non-training peers					Test result	*p* *
	*N*	Mean	Min.	Max.	*SD*	*N*	Mean	Min.	Max.	*SD*		
SP (mm)	21	313.10	251.00	385.00	38.05	80	343.09	198.00	599.00	81.34	C-C = −2.44	0.017
MA (mm)	21	1.23	0.50	3.20	0.61	80	2.71	0.80	5.60	1.09	*U* = −5.82	0.000
SA (mm^2)	21	113.43	36.00	305.00	61.24	80	303.16	75.00	746.00	152.25	*U* = −5.88	0.000
MF (Hz)	21	1.60	0.60	2.91	0.61	80	0.77	0.30	1.75	0.31	*U* = 5.49	0.000

*p–significance level; C-C, value of the Cochran–Cox test; *U*–value of the Mann–Whitney U test.

In the older age group, significant intergroup differences were noted for all analyzed indicators (Table 4). In the case of SP (mm), MA (mm), and SA (mm^2), higher averages were recorded for those who did not train, and in the case of MF (Hz), a higher average was recorded for judo competitors.

**TABLE 4:** Values of parameters characterizing the level of balance of adolescents (13–14 years old) practicing judo and their non-training peers.

	Adolescent judokas					Non-training peers					Test result	*p*
	*N*	Mean	Min.	Max.	*SD*	*N*	Mean	Min.	Max.	*SD*		
SP (mm)	18	257.83	190.00	326.00	42.54	76	312.71	166.00	470.00	75.93	CC = −4.1	0.000
MA (mm)	18	1.62	0.60	3.50	0.88	76	2.23	0.90	5.50	0.84	*U* = −2.9	0.003
SA (mm^2)	18	118.50	34.00	240.00	69.10	76	230.79	69.00	594.00	114.62	U = −4.2	0.000
MF (Hz)	18	1.07	0.48	2.11	0.51	76	0.82	0.35	1.56	0.27	CC = 2.1	0.049

C-C, value of the Cochran-Cox test; *U*–value of the Mann–Whitney U test.

Comparing the means between children judokas and adolescent judokas, significant differences were noted in the case of SP (mm) and MF (Hz). In both cases, higher mean values were observed in children, 331.10 and 1.60, respectively. In the group of youngsters, the mean SP (mm) was 257.83 and MF (Hz) was 1.07 (Table 5).

**TABLE 5:** Values of parameters characterizing the level of balance in children and adolescent judokas.

	Children judokas					Adolescent judokas					Test result	*p*
	*N*	Mean	Min.	Max.	*SD*	*N*	Mean	Min.	Max.	*SD*		
SP (mm)	21	313.10	251.00	385.00	38.05	18	257.83	190.00	326.00	42.54	*t* = 4.28	0.000
MA (mm)	21	1.23	0.50	3.20	0.61	18	1.62	0.60	3.50	0.88	*U* = −1.35	0.176
SA (mm^2)	21	113.43	36.00	305.00	61.24	18	118.50	34.00	240.00	69.10	*U* = 0.03	0.978
MF (Hz)	21	1.60	0.60	2.91	0.61	18	1.07	0.48	2.11	0.51	*t* = 2.89	0.006

*t*, Student’s *t*-test value for unrelated variables; *U*–value of the Mann–Whitney U test.

A similar comparison in the group of non-training persons indicates statistically significant better values of all analyzed indicators in the group of adolescents (Table 6), with the exception of the frequency of MF (Hz) correction reactions.

**TABLE 6:** Values of parameters characterizing the level of balance in children and adolescents.

	Children					Adolescents					Test result	*p*
	*N*	Mean	Min.	Max.	*SD*	*N*	Mean	Min.	Max.	SD		
SP (mm)	80	343.09	198.00	599.00	81.34	76	312.71	166.00	470.00	75.93	*t* = −2.4	0.017
MA (mm)	80	2.71	0.80	5.60	1.09	76	2.23	0.90	5.50	0.84	*U* = −2.9	0.004
SA (mm^2)	80	303.16	75.00	746.00	152.25	76	230.79	69.00	594.00	114.62	*U* = 3.0	0.003
MF (Hz)	80	0.77	0.30	1.75	0.31	76	0.82	0.35	1.56	0.27	*U* = −1.3	0.180

*t*, Student’s *t*-test value for unrelated variables; *U*–value of the Mann–Whitney U test.

When examining the relationship between the values of the parameters characterizing the balance level and the sports level, both in the group of children and adolescents, insignificant values of correlation coefficients were obtained (Tables 7, 8).

**TABLE 7:** Values of rank correlation coefficient calculated between the parameters characterizing the level of balance and the level of achievement in competitions in the group of children.

Correlated parameters	*N*	*R*	*t*(*N*-2)	*p*
Level of achievement and SP	21	-0.099	-0.434	0.669
Level of achievement and MA	21	0.113	0.494	0.627
Level of achievement and SA	21	0.093	0.408	0.688
Level of achievement and MF	21	-0.180	-0.798	0.435

**TABLE 8:** Values of the rank correlation coefficient calculated between the parameters characterizing the level of balance and the level of achievement in competitions in the group of adolescents.

Correlated parameters	*N*	*R*	*t*(*N*-2)	*p*
Level of achievement and SP	18	0.296	1.241	0.232
Level of achievement and MA	18	0.174	0.708	0.489
Level of achievement and SA	18	0.278	1.158	0.264
Level of achievement and MF	18	-0.174	-0.705	0.491

## Discussion

The aim of this study was to analyze the impact of training and selection on balance indices in judo practitioners aged 11–14, and to determine their relationship with the level of performance in competitions.

The results obtained during the study indicate a positive influence of the trained discipline on the static balance indicators. Both in younger and older groups of judokas, a higher level of balance control mechanisms was found than in their non-training peers. The author’s own data are confirmed by numerous studies in which the authors note the positive effect of combat sports on improving the functioning of the vestibular organ, especially deep feeling, which in judo plays a major role in stability control (Lemoth et al., 2009; Fong et al., 2011; Truszczyńska et al., 2015; Maśliński et al., 2017). In the case of combat sports practitioners, where a significant part of the competition requires, for example, maintaining the balance in a one-legged position (similar to, e.g., dancers or skaters), the advantage over the non-training individuals is observed in all conditions of the balance task (static and dynamic), unilateral and bilateral (Hahn et al., 1999; Perrin et al., 2002; Matsuda et al., 2008). As one may also find data that do not confirm the occurrence of such relationships (Witkowski et al., 2014), a few remarks should be made: firstly, not all physical activity improves stability control mechanisms to the same extent; secondly, externalization of their efficiency may require certain conditions (static or dynamic); and thirdly, and finally, not all traditional methods of assessing balance, especially in static, are adequate for assessing its mechanisms in athletes.

The influence of the sports level on the stability of judokas is not explicitly assessed positively in the publications. There are studies in which the authors find no differences in the static balance indicators between athletes of various sports levels (Maśliński et al., 2015, 2016). At the same time, they notice significant differences between them in terms of dynamic balance. The reason for this may be the fact that judo training alters the hierarchy of receptor inputs used in balance control in favor of the increasing role of visual and vestibular information in elite athletes (Witkowski et al., 2004). A comparison of athletes at different stages of sports specialization shows that not always lower values of stability indicators represent its higher level (Witkowski et al., 2021). Based on the research, the authors note that athletes, under the influence of training (which is an expression of specific motor adaptations to specific tasks) acquire and train specific motor strategies. Therefore, they caution against the automatic unfavorable interpretation of the increased indexes of the displacement range of COP and the surface area SA. Our study fully supports this thesis, as lower values of the SP path length in older judokas are accompanied by a higher amplitude of corrective reactions (MA). This is justified by the increased sensitivity thresholds of sensory systems, which results in a delayed response increasing the economy of corrective reactions (Blaszczyk et al., 1993; Cieśliński et al., 2016, 2017; Witkowski et al., 2018). This is in line with the reduced frequency of these reactions (MF) discussed later. The increased swaying range observed in qualified judokas compared to beginners may therefore be a sign of increased adaptation. It is also significant to compare the differences in the frequency of corrective reactions between the non-trainers and judokas. In the former, an increase in frequency can be seen, which, in the light of the literature, indicates a lower economy of maintaining the balance. According to the authors, this applies to both free standing and more demanding positions (maximum lean, one-legged standing, etc.). The authors observe an increase in frequency alongside with the complication of a motor task (Kuczyński, 2000), therefore its lower values in qualified athletes are a direct evidence of more efficient performance of postural tasks.

The lack of correlation between the sports level indicators and balance indicators suggests the existence of more important components influencing the sport performance than a high level of balance. The lack of a relationship between the balance ability and the sports level of judokas was also noted by Hrysomallis (2011), indicating that balance training may be a factor in aiding athlete development by creating better conditions for mastering technical skills.

The conducted study has some limitations resulting primarily from the adopted methodology and the instrumentation used. Measuring balance in static conditions may not fully reveal differences between training and non-training individuals. Being a cross-sectional one, our study lacks the strength of the experiment, so we can only assume that the differences found are mainly the effect of post-training changes. It also seems that the natural direction of further work should be to determine changes in dynamic stability under the influence of controllable experimental stimuli.

## Conclusion

The analysis of the obtained results shows that in each category the trainers dominate over the non-trainers. This is a clear confirmation that practicing judo supports the natural processes of the development of postural stability by enhancing their effect.

Bearing in mind the limitations presented in the discussion section, it should be stated that in all the examined age categories, the training individuals are characterized by a higher level of stability. This trend overlaps with the natural process of improving stability indicators, also confirmed in the study. However, the hypothesis assuming a relationship between stability and sports level was not confirmed. In this case, it seems that it may be due to the wide range of the presented level, which, given the relatively small numbers, did not allow to capture any possible relationships. On the other hand, additional research would perhaps make it possible to determine whether the improvement of the competitive level of judokas from a certain level on depends to a greater extent on other elements than stability.

## Data availability statement

The original contributions presented in this study are included in the article/supplementary material, further inquiries can be directed to the corresponding author.

## Ethics statement

The studies involving human participants were reviewed and approved by the Regional Medical Chamber in Kraków, Poland, No. 108/KBL/OIL/2014. Written informed consent to participate in this study was provided by the participants’ legal guardian/next of kin.

## Author contributions

JJ and GL contributed to the conception and design of the study. JJ, GL, PB, and KS organized the database and performed the statistical analysis. JJ, GL, KW, PB, KS, and PP wrote the first draft of the manuscript. GL and PP wrote sections of the manuscript. All authors contributed to the article and approved the submitted version.
